# Early recovery following new onset anosmia during the COVID-19 pandemic – an observational cohort study

**DOI:** 10.1186/s40463-020-00423-8

**Published:** 2020-05-04

**Authors:** Claire Hopkins, Pavol Surda, Emily Whitehead, B. Nirmal Kumar

**Affiliations:** 1 Guy’s and St Thomas’ Hospitals, London, SE1 9RT UK; 2 King’s College, London, UK; 3 Newstead Wood School, Bromley, UK; 4 Edge Hill University Medical School, Ormskirk, UK

**Keywords:** COVID-19, Anosmia, Epidemiology, Olfactory dysfunction

## Abstract

**Background:**

A rapidly evolving evidence suggests that smell and taste disturbance are common symptoms in COVID-19 infection. As yet there are no reports on duration and recovery rates. We set out to characterise patients reporting new onset smell and taste disturbance during the COVID-19 pandemic and report on early recovery rates.

**Methods:**

Online Survey of patients reporting self-diagnosed new onset smell and taste disturbance during the COVID-19 pandemic, with 1 week follow-up.

**Results:**

Three hundred eighty-two patents completed bot an initial and follow-up survey. 86.4% reported complete anosmia and a further 11.5% a very severe loss of smell at the time of completing the first survey. At follow-up 1 week later, there is already significant improvement in self-rating of severity of olfactory loss. 80.1% report lower severity scores at follow-up, 17.6% are unchanged and 1.9% are worse. 11.5% already report compete resolution at follow up, while 17.3% report persistent complete loss of smell, with reported duration being 1 to over 4 weeks. This is reflected in the overall cumulative improvement rate of 79% patients overall in the interval between surveys.

**Conclusions:**

A review of the growing evidence base supports the likelihood that out cohort have suffered olfactory loss as part of COVID-19 infection. While early recovery rates are encouraging, long term rates will need to be further investigated and there may be an increase in patients with persistent post-viral loss as a result of the pandemic.

We further call for loss of sense of smell to be formerly recognised as a marker of COVID-19 infection.

## Background

Viruses that give rise to the common cold are well known to cause post-infectious loss, and post-viral anosmia is one of the leading causes of loss of sense of smell in adults accounting for up to 40% cases of anosmia [1]. Nasal respiratory epithelial cells and olfactory epithelial support cells have been shown to express high levels of ACE2 proteins used by the SARS-Cov2 virus, which causes the COVID-19 syndrome, to infect cells [2]. Previously described human strains of coronavirus have also been demonstrated to invade the central nervous system and propagate from within the olfactory bulb [3]. It is therefore perhaps no surprise that alterations in sense of smell would be reported by patients with COVID-19.

Early reports of anosmia occurring in association with COVID-19 appeared in the press; in Germany, one study found that more than 2 in 3 confirmed reported loss of smell and taste (https://www.faz.net/aktuell/gesellschaft/gesundheit/coronavirus/neue-corona-symptome-entdeckt-virologe-hendrik-streeck-zum-virus-16681450.html). In a study of 3191 COVID-19+ patients who were self-isolating at home with mild disease, 15.3% expressed smell or taste loss (https://news.joins.com/article/23738003?cloc=joongang-mhome-group6&fbclid=IwAR33__i-aKtLN2MzCs5AE-). Many anecdotal reports of increased incidence of anosmia widely shared on medical discussion boards by surgeons from all regions managing a high incidence of COVID-19 cases.

The authors similarly noted a sudden increase in anosmia in young, health and otherwise asymptomatic patients prompting the release of a press-release [4] aimed to highlight the potential link with COVID-19 to fellow ENT surgeons and encourage use of personal protective equipment (PPE) in such cases. Following the release of the statement, there has been considerable media attention and celebrities, politicians and many others have shared their recent onset of anosmia on social media – both in confirmed and unconfirmed COVID-19 cases. The first author received a deluge of emails from colleagues and patients reporting recent onset anosmia, seeking advice and volunteering help. These patients were asked to complete a survey regarding their symptoms. Here we present self-reported recovery rates and review the emerging peer-reviewed literature. 

## Methods

A survey to audit the onset of anosmia and associated symptoms was designed and sent to patients making contact by email for advice on their recent onset anosmia, along with an advice sheet [5]. The survey was initially conducted anonymously, and no reward was offered for completion. Many patients confirmed that they had completed the survey and also reported that they had shared the survey with multiple immediate family members, close friends and colleagues who were similarly afflicted. Within 24 h of sending approximately 300 email replies, 710 respondents had completed the survey. At 7 days, 2428 responses had been received, after sending a link to the survey in approximately 600 emails in response to patient queries, suggesting wider dissemination. The findings from this primary survey are reported elsewhere [6]. The database was checked for duplicate replies, but all completed surveys appeared unique. After confirmation that ethical approval was not required to allow patients wishing to participate in future studies to leave contact details, an optional box was added, 613 patients with known email contact details were contacted to complete a second survey 1 week after the completion of the first. Patients were allowed 72 h to complete the survey. Surveys were matched using email addresses, and then an anonymised database was used for analysis.

Descriptive data is presented. Tests for significance were performed using a Chi-Squared test and Mann Whitney U Tests.

## Results

**Fig. 1:**
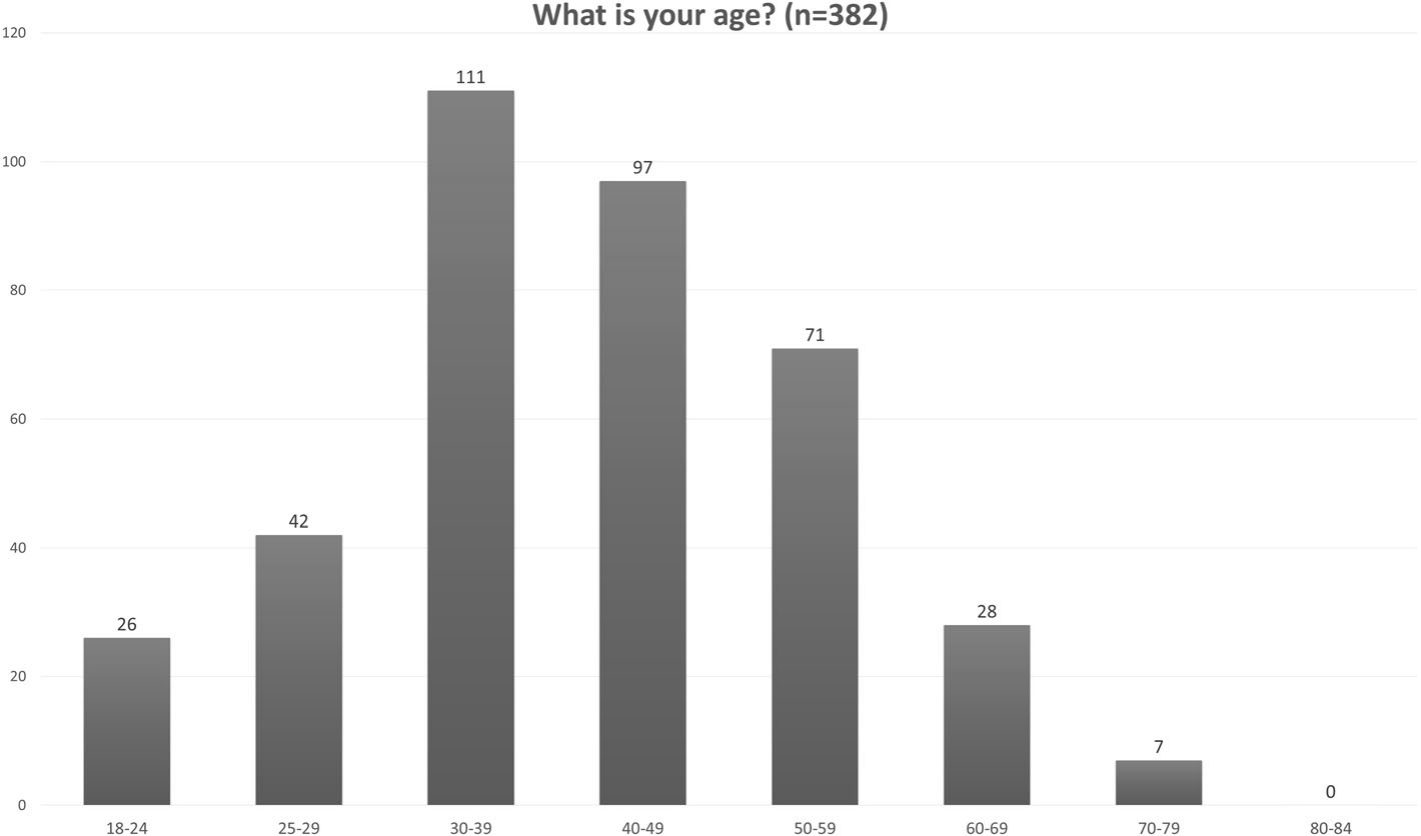
Age distribution of respondents

Three hundred eighty-two matched responses were received for the first and second survey (giving a response rate of 62.3%). Comparison to the larger cohort who completed the first survey suggests that non-responders were younger. In the initial cohort, 64% of 2428 respondents were aged under 40 and the median was 30 – 39 years (Fig. 1). 73% were female. In the current cohort of paired first and follow-up survey, 46.8% are under 40, the median age range is 40–49 years. 74.6% are female. Only 15 patients had been tested for COVID-19, but of those 80% tested positive for COVID-19.

**Fig. 2:**
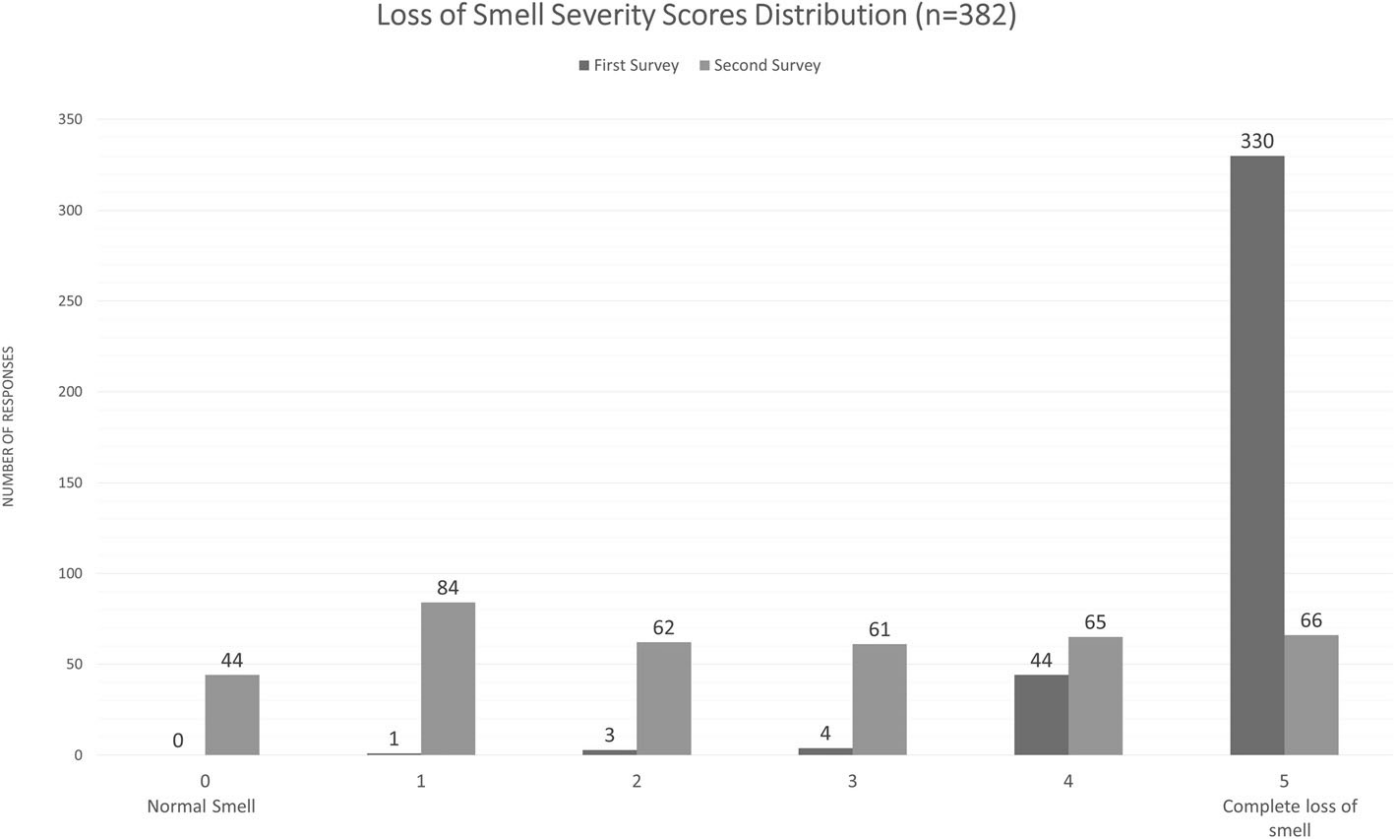
Severity rating of loss of sense of smell at first and second survey

**Fig. 3:**
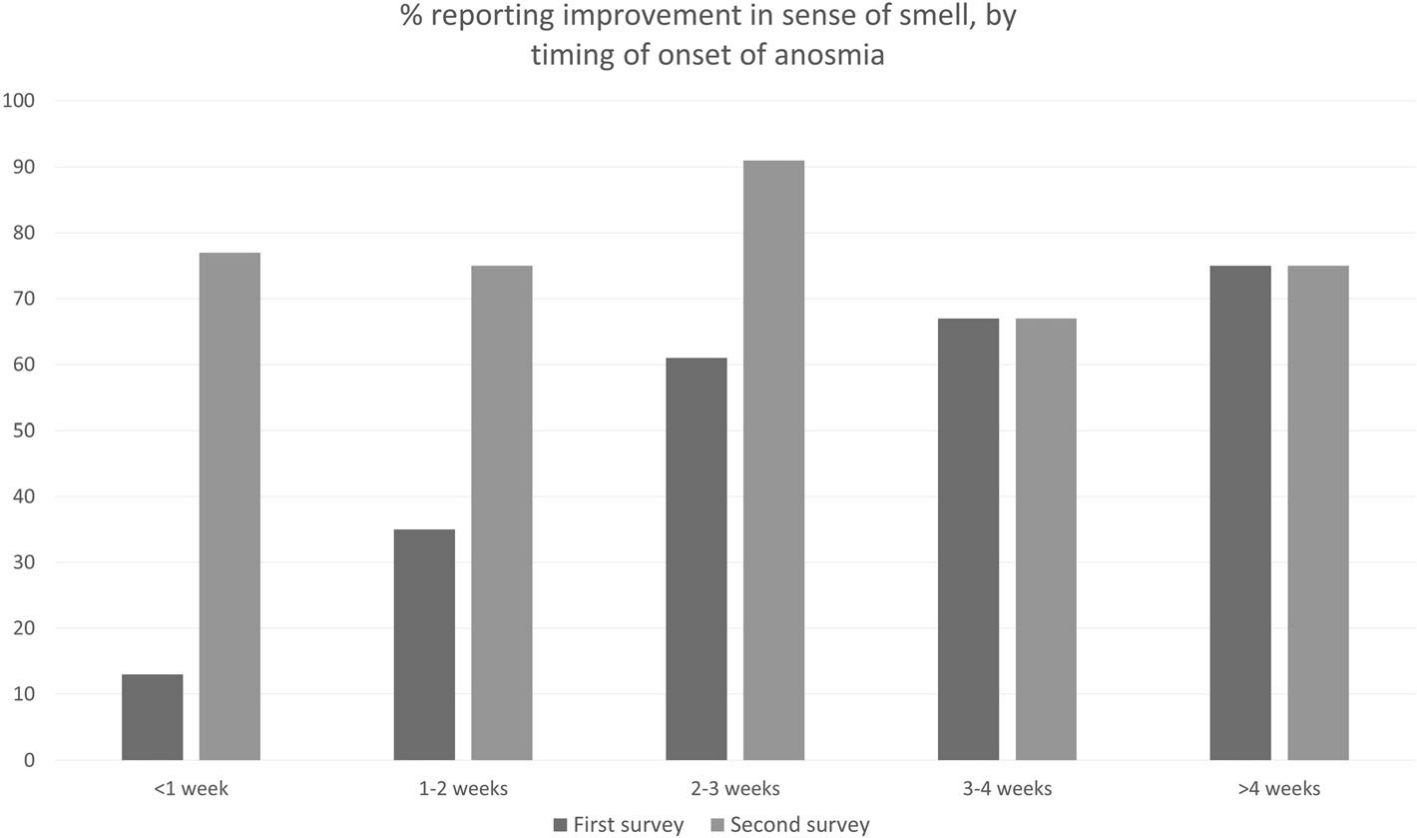
Self-reported improvement in sense of smell by time of onset, relative to completion of first survey

The majority of respondents (*n* = 229,60%) report onset of their anosmia less than 1 week before completing the first survey. One hundred twenty completed the first survey 1–2 weeks after onset, 26 at 2–3 weeks after onset, 3 at 3–4 weeks, and 4 more than 4 weeks after onset. 86.4% reported complete loss of sense of smell, a further 11.5% reported a very severe loss at the time of completing the first survey. At the time of completion of the second survey, there is significant improvement in self-rating of severity of olfactory loss (*P* < 0.001, Mann Whitney U 16131.5), (Fig. 2). 80.1% report lower severity scores at follow-up, 17.6% are unchanged and 1.9% are worse. 11.5% report no problem at all at follow up, while 17.3% report persistent complete loss of smell. This is reflected in the overall cumulative response rate of 79% patients overall stating that they had noticed improvement at the time of either the first or second survey. The time of reporting improvement in relation to the time of onset is shown in Fig. 3. At the first survey, only 13% of patients who had developed loss sense of smell in the preceding week had noticed improvement, compared with 35% 1–2 weeks after onset, 61% 2–3 weeks after onset, 67% for those between 3 and 4 weeks from onset, and 75% of those whose loss of sense of smell had first started more than 4 weeks earlier. There appears to be significant improvement in the first 2 weeks but then the recovery rate appears to plateau. Although the numbers are too small to make meaningful conclusions (*n* = 7), the overall recovery rate reported in those of 3 weeks duration or longer is 71%.

Sixty-nine patients reported accessing patient support groups for loss of sense of smell, and 36 reported using olfactory training (OT). The recovery rate in those using OT was 83% compared to 73% in those that did not perform OT, however the difference was not statistically significant (Chi2 1.71, *p* = 0.183).

Small numbers of patients tried other treatments (nasal steroids; 20 patients, omega 3 supplements; 11 patients, Vitamin A drops; 2 patients and Alpha lipoic acid supplements; 1 patient). Only 3 patients had been given oral steroids.

94.8% reported that their sense of taste was reduced at the first survey, but this had fallen to 75% at the second survey. 60% of the whole group reported that they could still differentiate between sweet, salty, sour and bitter tastes at the time of the first survey, improving to 88.9% at the second survey, suggesting the predominant issue is reduction in sense of smell rather than hypogeusia, although true taste disturbance should be considered. In free text, some patients also reported altered taste or a metallic taste, although we are not able to further determine if this reflects true dysgeusia from the survey.

At the time of completing the first survey, 14.9% who reported smell and /or taste disturbance did not report any other symptom thought to be associated with COVID-19. In patients who reported other symptoms, 14.9% reported anosmia before their onset, 39.3% at the same time, and in 45.8% the onset of anosmia came after other symptoms. At the time of the second survey, of the 57 patients who presented with isolated anosmia, 44 had subsequently developed other symptoms associated with COVID-19 infection, leaving isolated loss of sense of smell or taste in only 3.4% of the cohort.

## Discussion

When the authors released the original statement alerting ENT surgeons to the potential link between COVID-19 infection and smell and taste disturbance, there was no peer-reviewed literature. Despite growing anecdotal evidence to support a link with COVID-19 infection, there is still a paucity of peer-reviewed literature to support a causal association. At the time of writing, there are a number of letters available in Medline, repeating the largely anecdotal reports. A small case-series of patients seen by the primary author [7], prompting the original press-release, and the initial results of the survey have been published [6], reporting on 2428 patients presenting with recent onset anosmia during the COVID-19 pandemic, of whom 16% report loss of sense of smell as an isolated symptom. However, the majority of patients described in our series are not eligible for testing, which is currently only available in the UK for patients hospitalised with severe disease. Mao et al. described neurological manifestations of hospitalised patients with COVID19 in Wuhan, where hypogeusia and hyposmia were reported by 5.6 and 5.15% of patients respectively [8].

Lechien et al. [9] are the first peer-reviewed multicentre study, reporting on a series of 417 confirmed mild-moderate patients; 85.6 and 88.0% reported olfactory and gustatory dysfunction, respectively, with olfactory dysfunction emerging before other symptoms in 11.8% cases. Kaye et al. report on 237 US patients with COVID-19 and found that 73% reported anosmia, and that loss of sense of smell was the initial symptom in 26.6% [10].

There are a growing number of papers emerging on pre-print servers; Giacomelli et al. interviewed 59 hospitalised COVID-19 patients and found 34% reported smell or taste disturbance [11]. Females more frequently reported smell or taste disturbance than males, and patients with smell and taste disturbance were younger than those without.

Subsequent to initial reports suggesting anosmia could be a symptom of COVID-19, loss of sense of smell was added to a symptom tracker app developed by a team at King’s College London, with 1.5 million app users between 24 and 29 March 2020. Of these, 1702 reported having been tested for COVID-19, with 579 positive results and 1123 negative results. 59% of COVID-19 positive patients reported loss of smell and taste, compared with only 18% of those who tested negative [12]. These results were much stronger in predicting a positive COVID-19 diagnosis than self-reported fever. Using all the data collected, the King’s research team developed a model to identify which combination of symptoms together could predict COVID-19 cases which include a combination of loss of smell and taste, fever, persistent cough, fatigue, diarrhoea, abdominal pain and loss of appetite. The strongest predictor is loss of smell and taste. In a similar study, Yan et al. found smell and taste lost were reported by 68 and 71% of COVID positive patients respectively, compared to 16 and 17% of COVID negative patients, with loss of sense of smell being the strongest predictor (OR 10.9 for COVID-19 positivity) [13].

Importantly, virological assessment of patients with mild COVID-19 symptoms, including smell and taste disturbance in some patients, have confirmed high levels of viral shedding, which confirm that these patients may indeed spread disease and that self-isolation should be recommended [14].

An apparent female predominance found in our study and in that of Lechien must be interpreted with caution, as it may simply reflect gender differences in completing voluntary questionnaires. However, this is also supported by the study of Giacomelli et al. The literature also suggests higher prevalence of anosmia in mild-moderate disease versus severe disease, and in younger age groups. We cannot attribute any prognostic significance to the presence of anosmia without widespread epidemiologic study across large populations, but there do appear to be 2 different phenotypes of COVID-19 disease; those with mild disease where anosmia is prevalent, found more often in younger and female patients, and those with moderate to severe respiratory disease, seen more frequently in older and male patients. It might be that smell and taste disturbance simply goes unreported in the presence of respiratory distress.

Of particular interest in our first survey was the 1 in 6 patients who have developed anosmia in the absence of any other symptoms. At the follow-up survey, most of these patients had developed other symptoms, but only 50% reported a cough or fever across either survey. Overall, nearly 1 in 4 respondents reported anosmia as an isolated symptom, or as the first presenting symptom. Using current World Health Organisation and Public Health England definitions, half of the participants of this survey would not currently be suspected to have COVID19 and may be some of the hitherto hidden carriers that have facilitated the rapid spread of infection, as they do not meet criteria where self-isolation would be recommended. Formal recognition of loss of smell and taste as a marker of infection, to allow identification of paucisymptomatic patients is critical to reduce transmission when capacity for formal testing is limited.

This is the first reported study to report follow-up data – albeit of very short duration. Our study shows that nearly 80% of patients report improvement in loss of sense of smell within a few weeks of onset, with recovery appearing to plateau after 3 weeks. Lower rates of spontaneous recovery have been previously been reported with post-viral anosmia, ranging from 32% [15] to 67% [16] in the absence of any active treatment. It is likely that many of cases of loss of sense of smell of short duration would normally go unreported, and only patients with symptoms of longer duration would seek medical advice. The 71% recovery rate reported in those of duration of 3 weeks or more is more in keeping with previously reported studies. Long-term studies of COVID-19 survivors are required, as well research to determine for how long patients may remain infectious.

It appears that otolaryngologists may expect a surge in patients with loss of sense of smell when elective medical care resumes after the pandemic. Hopefully, patients will be seen after a period where viral shedding has ceased, but appropriate personal protective equipment will still be required if seeing cases of new onset. Nasal endoscopy should not be performed within the first 2 weeks of onset, unless there are other red-flag symptoms (notably, blood stained discharge). Although a number of different treatments have been described almost all trials reported have very small numbers, most lack a blinded, randomised, control arm and rates of improvement are usually no greater than reported rates of spontaneous improvement. A meta-analysis of 13 studies of olfactory training suggests small to moderate benefit [17]; we found no difference in early improvement rates in our study, however numbers using OT were very small, and training over at least 12 weeks is recommended. Current WHO guidance (https://www.who.int/docs/default-source/coronaviruse/clinical-management-of-novel-cov.pdf) is to avoid the use of systemic steroids in cases with or at risk of severe acute respiratory COVID-19 as systematic review suggests possible harm including delayed viral clearance; this recommendation appears to have been well observed with only 3 patients reporting having taken them.

The major limitation of this study is the lack of confirmed COVID-19 status. The majority of our cohort do meet current criteria for testing in the UK, as it is only being undertaken in hospitalised patients, however, of those tested in our survey, 80% were found to be positive, While we do not know the means of testing, but in most cases, RT-PCR analysis of nasal swabs has been used, which have an estimated sensitivity of 72% [18]. Many patients also reported other symptoms described in COVID-19 infection, with half also reporting a new onset cough or fever, therefore making COVID-19 likely according to the results of Menni et al. [12] and Yan et al. [13]. However, our assumption that our cohort reflects patients with COVID-19 cannot be proven without test results even though it is supported by the evince base. Post-viral anosmia is a common cause of olfactory loss, and transient loss is likely under-reported such that the true incidence is unknown. Our cases may have been caused by unrelated rhinovirus and coronavirus strains, that would have previously gone unreported, were it not for the attention COVID19 has attracted in the media. We cannot, therefore estimate the overall prevalence of anosmia in those infected with COVID-19. However, until widespread testing in the community is undertaken, any study in only those with confirmed COVID-19 to also prone to selection bias in evaluating a more severely affected, and likely older cohort requiring hospital admission. The benefit of this study is that it may offer a picture of patients who would likely be missed by current diagnostic criteria, and studies of selected populations with severe disease requiring hospitalisation or targeted testing regimens.

We are also unable to verify the self-reported reduction in sense of smell or subsequent improvement with objective testing or review of medical records. 

## Conclusions

While further research is still required, we believe the current study and the rapidly evolving evidence base supports an urgent need to add anosmia to the list of symptoms used in screening tools for possible COVID-19 infection; it is untenable for public health organizations to disregard this symptom any longer. Use of loss of smell and taste as a marker for infection will be a very useful weapon in the COVID-19 fight, especially in countries where access to testing will be greatly limited. For those who have lost their sense of smell and taste during the COVID-19, we can offer reassurance that recovery is likely in the majority of cases.

## Data Availability

The datasets used and/or analysed during the current study are available from the corresponding author on reasonable request.

## References

[1] Welge-Lussen A, Wolfensberger M (2006). Olfactory disorders following upper respiratory tract infections. Adv Otorhinolaryngol.

[2] Brann D TT, Weinreb C, Logan D, Datta S. Non-neural exporessio of SARS-CoV-2 entry genes in the olfactory epithelium suggests mechanisms of underlying anosmai in COVID-19. Biorxiv.org; 2020. 10.1101/2020.03.25.009084.

[3] Dube M, Le Coupanec A, Wong AHM, Rini JM, Desforges M, Talbot PJ. Axonal Transport Enables Neuron-to-Neuron Propagation of Human Coronavirus OC43. J Virol. 2018;92(18). 10.1128/JVI.00404-18. 10.1128/JVI.00404-18 PMC6096804 29925652

[4] Hopkins C, Kumar N (2020). Loss of sense of smell as a marker of COVID-19 infection.

[5] Hopkins CGS, Philpott C (2020). Advice for patients with recent onset loss of smell during the COVID-19 pandemic.

[6] Hopkins C, Sruda P, Kumar N. Presentation of new nset anosmia during the COVID-19 pandemic. Rhinol. 2020. 10.4193/Rhin20.116. 10.4193/Rhin20.116 32277751

[7] Gane SB, Kelly C, Hopkins C. Isolated sudden onset anosmia in COVID-19 infection. A novel syndrome? Rhinol. 2020. 10.4193/Rhin20.114. 10.4193/Rhin20.114 32240279

[8] Mao L WM, Chen S, He Q, Chang J et al. Neurological manifestations of hospitalised patients with COVID-19 in Wuhan China: a retrospective case series. Preprint on medrxivorg. 2020.

[9] Lechien JR, Chiesa-Estomba CM, De Siati DR, Horoi M, Le Bon SD, Rodriguez A, et al. Olfactory and gustatory dysfunctions as a clinical presentation of mild-to-moderate forms of the coronavirus disease (COVID-19): a multicenter European study. Eur Arch Otorhinolaryngol. 2020. 10.1007/s00405-020-05965-1 PMC7134551 32253535

[10] Kaye R CC, Kazahaya K, Brereton J et al. COVID-19 anosmia reporting tool: initial findings. Accepted preprint for Oto-HNS. 2020. 10.1177/0194599820922992 32340555

[11] Giacomelli A PL, Conti F, Bernacchia D, Siano M et al. Self-reported olfactory and taste disorders in SARS-CoV-2 patients: a cross-sectional study Clinical Infectious Diseases 2020, preprint 10.1093/cid/ciaa330. 10.1093/cid/ciaa330 PMC7184514 32215618

[12] Menni C VA, Freydin M, Ganesh S et al. Loss of smell and taste in combination with other symptoms is a strong predictor of COVID-19 infection. MedRxiv preprint server. 2020.

[13] Yan CH, Faraji F, Prajapati DP, Boone CE, DeConde AS. Association of chemosensory dysfunction and Covid-19 in patients presenting with influenza-like symptoms. Int Forum Allergy Rhinol. 2020. 10.1002/alr.22579 PMC7262089 32279441

[14] Wolfel R, Corman VM, Guggemos W, Seilmaier M, Zange S, Muller MA, et al. Virological assessment of hospitalized patients with COVID-2019. Nature. 2020. 10.1038/s41586-020-2196-x 32235945

[15] Reden J, Mueller A, Mueller C, Konstantinidis I, Frasnelli J, Landis BN (2006). Recovery of olfactory function following closed head injury or infections of the upper respiratory tract. Arch Otolaryngol Head Neck Surg.

[16] Duncan HJ, Seiden AM (1995). Long-term follow-up of olfactory loss secondary to head trauma and upper respiratory tract infection. Arch Otolaryngol Head Neck Surg.

[17] Sorokowska A, Drechsler E, Karwowski M, Hummel T (2017). Effects of olfactory training: a meta-analysis. Rhinology.

[18] Wang W, Xu Y, Gao R, Lu R, Han K, Wu G, et al. Detection of SARS-CoV-2 in different types of clinical specimens. JAMA. 2020. 10.1001/jama.2020.3786 PMC7066521 32159775

